# Targeted Therapy of Hepatitis B Virus-Related Hepatocellular Carcinoma: Present and Future

**DOI:** 10.3390/diseases4010010

**Published:** 2016-02-15

**Authors:** Sarene Koh, Anthony Tanoto Tan, Lietao Li, Antonio Bertoletti

**Affiliations:** 1 Singapore Institute for Clinical Sciences, Agency for Science, Technology and Research (A*STAR), Singapore 117609, Singapore; sarene_koh@sics.a-star.edu.sg; 2 Lion TCR Private Limited Singapore, Singapore 069113, Singapore; lietao.li@gmail.com; 3 Emerging Infectious Diseases (EID) Program, Duke-NUS Graduate Medical School, Singapore 169857, Singapore; anthony.tan@duke-nus.edu.sg

**Keywords:** immunotherapy, liver tumor, HBV, T cell engineering

## Abstract

Cancer immunotherapy using a patient’s own T cells redirected to recognize and kill tumor cells has achieved promising results in metastatic melanoma and leukemia. This technique involves harnessing a patient’s T cells and then delivering a gene that encodes a new T cell receptor (TCR) or a chimeric antigen receptor (CAR) that allow the cells to recognize specific cancer antigens. The prospect of using engineered T cell therapy for persistent viral infections like hepatitis B virus (HBV) and their associated malignancies is promising. We recently tested in a first-in-man clinical trial, the ability of HBV-specific TCR-redirected T cells to target HBsAg-productive hepatocellular carcinoma (HCC) and demonstrated that these redirected T cells recognized HCC cells with HBV–DNA integration [1] We discuss here the possibility to use HBV-specific TCR-redirected T cells targeting hepatitis B viral antigens as a tumor specific antigen in patients with HBV-related HCC, and the potential challenges facing the development of this new immunotherapeutic strategy.

## 1. Introduction

There are limited therapeutic options for hepatocellular carcinoma (HCC), the third leading cause of cancer deaths worldwide [2]. Liver transplantation still represents the better treatment option, but advanced cases are not eligible and, despite stringent selection criteria, HCC relapses still occur in approximately 20% of transplanted patients [3]. Surgical resection is also an option for small tumors but survival at five years is, in any case, bleak. Up to 70% of patients experience HCC recurrence within two years after primary hepatic resection, and only 10% of patients survive more than three years [3]. There is, therefore, a real necessity to find new efficient treatments for this disease. In this manuscript, we will discuss the possibility to use immunotherapeutic strategy based on T-cell receptor engineered T cells targeting HBV antigen in the treatment of HBV-related HCC.

## 2. T Cell Immunotherapy in Tumors

**Figure 1:**
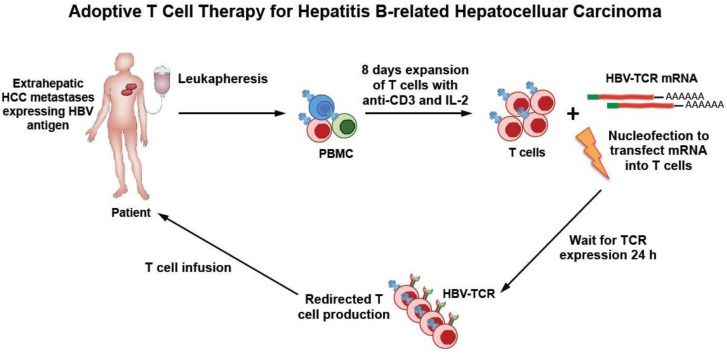
A schematic illustrating production of HBV-TCR redirected T cells using messenger RNA electroporation, followed by adoptive transfer in a patient with HBV-related HCC. A patient’s own T cells can be expanded *in vitro* for eight days using soluble anti-CD3 antibody and high dose IL-2. The primary T cells are electroporated with mRNA encoding HBV-specific TCR to transiently redirect their specificity towards HBV. At 24 h post-electroporation, the T cells are analyzed for TCR expression using HLA class I pentamers specific for the introduced TCR, and the functionality of the redirected T cells are assessed (by their ability to produce IFN-g and degranulate upon antigen-specific stimulation). The redirected T cells can be re-infused into the patient, whereby they will recognize and lyse HCC cells expressing HBV antigen within 3–4 days and then their specificity is lost.

Cytotoxic T lymphocytes, our body’s natural serial killers, play an important role in killing cancerous and infected cells. They express T cell receptors that recognize a specific antigen that is produced intracellularly by pathogens or cancer cells. The intracellular pathogen or tumor proteins are processed and presented as peptide fragments on major histocompatibility complex (MHC) class I molecules on the surface of the cells and the ensuing MHC-class I/peptide complex is recognized by a particular specific T cell receptor (TCR) [4]. Once cytotoxic T cells recognize their target, they promote cell death of the target cell through a combination of cytotoxins and receptor-mediated mechanisms [4]. The potent killing ability of cytotoxic T lymphocytes can be harnessed as mediators of antitumor immunity. Early immunotherapy using autologous cytokine activated killer cells showed some contribution to tumor regression or patient survival [5]. Later on, *in vitro* studies showed that human tumor-infiltrating lymphocytes obtained from resected melanomas contained specific T cells capable of recognition of autologous tumors *in vitro* [6], and the first successful adoptive cell therapy in patients with metastatic melanoma achieved objective regression of cancer [7]. However, the isolation and expansion of pre-existing tumor-specific T cells from most cancer patients is extremely difficult and time consuming. To overcome these limitations, gene transfer based strategies have been developed to retarget patients’ circulating lymphocytes to a chosen tumor antigen or tumor neo-antigen expressed by the cancer cells. This is achieved by the transfer of a chimeric antigen receptor (CAR) composed of antibody binding domains fused to T cell signaling domains or the transfer of TCR αβ heterodimers, and these genetic modifications rapidly endowed T cells with a defined antigen-specificity [8]. The main difference between chimeric (CAR) and classical T cell receptor is their specificity and MHC-class I restriction [9]. Since CAR specificity is instructed by the variable region of a specific antibody, CAR-T cells recognize conformational antigen expressed on the surface of the target cells (Figure 1). This recognition is not restricted by antigen presentation to selected MHC-class I or class II molecules and thus CAR-T cells can be used in all patients, irrespective of their MHC profile. In contrast, the TCR does not recognize whole antigens but short fragments of it (called epitopes) presented on the surface of cells by MHC-class I molecules and derived from proteins synthetized within the cells. TCR-redirected T cells can therefore recognize cells producing tumor or viral antigens, but since they are MHC-class I restricted, they can be used only in patients sharing MHC-class I alleles.

Importantly, adoptive T cell therapy using both chimeric antigen receptor- [10] and TCR- [11,12,13] engineered T cells have shown highly promising results in the treatment of several cancers, including melanoma and leukemia. The therapeutic potential of this therapy is now evaluated in several cancers.

## 3. Rationale of HBV-Specific T Cell Therapy in HBV-Related HCC

HCC development is associated with chronic liver inflammation. In Asia, where the incidence of HBV infection is high, at least 80% of HCCs are associated with HBV. HBV not only causes chronic liver inflammation, but, during the infection, it can integrate into the genome of hepatocytes [2,14]. A high frequency of HBV integrations (>80%) is observed in HBV-related HCC tumors [14]. This HBV–DNA integration can not only trigger oncogenic transformation of normal hepatocytes but can also result in the expression of HBV antigens in HCC cells [15]. High levels of HBV mRNA were also detected in about 30% of the HCC analyzed in a recent work [16].

Whether these specific features can be exploited for T cell immunotherapy in HCC has been the interest of our recent research work. In particular, we hypothesized that HBV-specific TCR can recognize the HBV antigens expressed on HCC cells, derived from the transcription of the integrated HBV–DNA [17]. The targeting of viral non-self antigens expression on HCC cells (like HBs or HBx antigens) can have advantages over the targeting of classical tumor-associated antigens.

HCC can express tumor associated antigens ((like NY-Eso-1, glypican-3, alpha-feto protein, Mage-3), and T cells specific for these tumor-associated antigens are present in HBV-related HCC patients [18], and they correlate with slower disease progression [19]. T cell receptor engineered T cells specific for glypican-3 have been recently developed [20]. However, the use of T cells engineered with TCR specific for classical tumor antigens can have drawbacks. First, T cell receptors specific for tumor-associated antigens are of low affinity and require modifications in order to efficiently recognize tumor cells. Such modifications can, however, lead to severe side effects due to the targeting of normal tissue [21] that can express self-tumor antigens at low levels. Alternatively, the expression of low quantity of tumor associated antigens in normal tissue can induce a state of tolerance in tumor-specific T cells [22].

In contrast, T cells recognizing HBV antigens present in patients that resolved acute HBV infection express high affinity T cell receptors, and we demonstrated that these HBV-specific TCRs expressed on T cells can generate new HBV-specific T cells able to lyse not only HBV infected cells but also natural HCC lines with HBV–DNA integration [23] both *in vitro* and in animal models [24]. Since HBV-specific T cell response is profoundly compromised in HCC patients [18,19], the adoptive transfer of HBV–TCR redirected T cells in patients with HBV-related HCC should reconstitute the deleted repertoire of HBV-specific T cells and target the HBV-expressing HCC cells. Furthermore, our TCR-redirected T cells are engineered with classical HLA-class I restricted TCR, hence, they are specific for HBV-peptide complexes presented on the surface of HCC cells and are not inhibited by the high quantity of circulating HBsAg present in HBV-infected patients’ sera [23]. This possibility might instead affect HBV-specific CAR-T cells [25], in whom the chimeric TCR (CAR) is specific for HBsAg. The selection of HBV antigen as a target for immunotherapy of HBV-related HCC had, however, one important drawback: in patients with chronic HBV infection who developed HCC, HBV antigens are expressed not only by HCC cells with HBV–DNA integration but also by HBV infected normal hepatocytes. The risk of inducing severe liver damage due to the targeting of HBV infected hepatocytes by HBV–TCR redirected T cells cannot be ignored [26]. We have very limited knowledge about a possible difference between the ability of normal and transformed hepatocytes to express, process and present HBV antigens, and, therefore, we don’t know whether HBV–TCR redirected T cells will be able to target preferentially HBV-expressing HCC cells or normal HBV infected hepatocytes [17]. This is the main reason why the first use of HBV–TCR-redirected T cells in clinic was performed in a unique clinical scenario with a much reduced risk of potential damage to normal liver tissue: a liver transplanted patient who develops extrahepatic HCC metastasis.

## 4. HBV–TCR Therapy in Liver Transplanted Patients with HCC Relapse

HBV–TCR redirected T cells were first used in a liver transplant patient who developed extrahepatic HCC metastasis with HBV–DNA integration producing HBsAg while complete HBV–DNA, a sign of HBV replication was absent in his blood and in the transplanted liver [1].

In this patient, we were first able to demonstrate that his extrahepatic HCC cells present HBV antigen in a conformation recognizable by HLA-class I restricted TCR since they express on the surface complex HLA-A0201/HBs183-91 complexes. The presence of HLA-A0201/HBV peptide complexes on the surface of cells present in extrahepatic HCC metastasis was tested using a T cell receptor like antibody specific for the complex HLA-A0201/HBs183-91 [27]. The expression of HBV-peptide complexes was homogeneously present in all the HCC cells derived from the metastasis demonstrating directly *in vivo* that HCC cells were not only producing HBV surface protein but were able to process and present on their surface HLA-A0201/HBV peptide complexes that can be recognized by engineered HBV-T cells.

A specific TCR targeting the complexes HLA-A0201/HBs183-91 was therefore used to engineer HBV–TCR specific T cells under good manufacturing practice for use in a compassionate, special license treatment. A retroviral vector was used to transduce autologous *in vitro* activated T cells. The patient received a single dose of ~1.2 × 10^4^ TCR-redirected T cells per kg—a low dose in comparison with the ones used in immunotherapy of other tumors but similar to doses used in anti-viral cell therapies [28]. The patient’s condition was very compromised at the time of the treatment, with disseminated HCC metastasis in lung, bones and neck, but the cell infusion was very well tolerated. There were no signs of acute toxicities and in the 30 days of observation period after infusion we detected only a minimal alteration of alanine aminotransferase (ALT) levels. Other important observations were derived from this first treatment: a) despite the lack or pre-emptive lymphodepletion or and cytokine therapy, TCR-redirected T cells efficiently expanded in the patient. A high frequency of TCR-redirected T cells (~2% total CD8 T cells) was detected 30 days after infusion demonstrating the ability of TCR-redirected T cells to expand *in vivo*. This is somehow surprising since HBV-specific CD8 T cell in patients with chronic hepatitis B (CHB) infection or in HBV/HCC patients are not only present at very low frequency (~0.01%/0.03% CD8 cells) but are functionally impaired [18]. Lack of functionality has been also been observed in tumor antigen specific T cells present in HCC patients [19]. In contrast, the HBV-TCR-T cells transferred in the patient not only proliferate but seem to function *in vivo*, since HBsAg levels dropped dramatically (3200 IU before therapy to ~300 IU at day 30) in concomitance with the HBV-TCR-T cells expansion. It is clear that we cannot derive any hard conclusion from data analysis derived from a single patient, but, at least in this case of extrahepatic HCC, the HBV antigen can trigger a robust T cell expansion and is not causing the deletion or tolerization of HBV-specific T cells observed in CHB patients. Regrettably, the robust HBsAg drop was not associated with any detectable reduction of the volume of the HCC metastasis. One month after T cell therapy, the general conditions of the patient declined and at week 8, brain metastasis was detected that resulted in confusion, lethargy and death nine weeks after therapy. Unfortunately, for cultural reasons, an autopsy was not performed to gather information relative to possible T cell infiltration in the HCC metastasis.

It is clear that more data needs to be gathered to understand the clinical efficacy of HBV-specific TCR redirected T cells in HCC patients. HBV-specific TCR cells can eradicate HCC tumors in SCID mice [24], but the multiple HCC locations present in this patient could represent a tumor burden that is too advanced to test the HBV-TCR T cells therapeutic efficacy.

One other question connected to the clinical efficacy of HBV-TCR T cells is whether their therapeutic use should be selectively applied to cases of HCC relapses in liver transplanted patients.

Since HCC relapses after liver transplant can be very high (about 50%—5 years in patients with liver transplants after HCC resection [29]) and serum HBsAg recurrence is associated with HCC recurrence (7/8 patients, 87.5%); one possibility is to use HBV-TCR T cells not only as a therapy but as a prophylactic treatment to prevent HCC seeding in liver transplanted patients with HBsAg positive HCC. In animal models, HCC seeding was blocked with the use of HBV–TCR-T cells [24].

Instead, as we have already pointed out, an obstacle for the implementation of TCR-therapy in CHB patients with primary HCC is our limited knowledge of whether level of expression, presentation and processing of HBV antigen differs in transformed or normal hepatocytes [17]. Research efforts are now increasing in this area and might lead to more clear answer to this possibility. We have, however, developed practical methods to reduce the potential toxicities of adoptive transfer of TCR-redirected T cells through production of HBV-specific T cells with mRNA electroporation and not with the use of stable retro- or lentiviral vectors (Figure 1).

Exogenous TCR can be expressed in lymphocytes using mRNA electroporation. Electroporation of mRNA encoding HBV-TCR resulted in expression 24 h after electroporation with a median receptor expression of 80.0% in activated T cells and with excellent cell viability. The TCR expressed through mRNA electroporation in lymphocytes is transient (3–4 days) [24], but, despite such transient expression, the mRNA TCR electroporated cells suppress HCC growth and stop HCC seeding in animal models [24]. The transient nature of mRNA HBV TCR expressing T cells will clearly reduce its potential toxicity and avoid the unchecked expansion of TCR-redirected T cells in patients. mRNA electroporation can therefore allow us to test clinical efficacy and toxicities of graded numbers of such cells that, differently from T cells engineered to stably express TCR through viral vectors, will be adoptively transferred over time and will not accumulate or expand in the body of the treated patients.

Furthermore, mRNA TCR is considerably easier and faster to be produced and do not carry the safety concerns connected with the integration and subsequent oncogenic potential of viral vectors [30] and thus can also be used as a possible prophylactic treatment of HCC relapses after liver transplantation.

## 5. Conclusions

We think that adoptive transfer of HBV–TCR redirected T cells in patients with HBV-related HCC represent a new therapeutic possibility that needs to be properly tested in HCC patients. Certainly the methods to produce and expand HBV–TCR redirected T cells for therapy are still experimental and not easily transferable to all hospitals. The quantity and frequency of TCR-redirected T cells to adoptively transfer will have to be carefully studied to find the correct treatment schedule. We also need to expand our TCR libraries in order to produce an array of TCRs able to recognize different HBV antigens restricted by different HLA-class I molecules that can be used in patients with different HLA-class I profile and HBV expression. Initial therapeutic tests will be performed in liver transplanted patients developing HCC relapses since the HLA-mismatched between the liver transplant and the HCC relapses originated from the primary tumor could allow the targeting of only the tumor cells and not the transplanted liver. This will exclude the possibility that our TCR-redirected T cells will recognize and kill normal HBV infected hepatocytes. Such a patient population is unfortunately not as rare as it might be though. Indeed, even after the adoption of stringent selection criteria, HCC relapses occur in approximately 20% of transplant patients [2]. Furthermore, direct analysis of HBV–DNA in these HCC cells have demonstrated HBV–DNA integration or full HBV replication [31,32,33] in most of them suggesting that a majority of these HCC relapses can indeed express HBV antigens and, as such, be possibly targeted by HBV-specific TCRs. Information gathered on this selected patients population will guide the development of this new immunotherapeutic strategy that might offer new hope of a cure of HCC.
